# Citizen-led expeditions can generate scientific knowledge and prospects for researchers

**DOI:** 10.1371/journal.pbio.3001872

**Published:** 2022-11-15

**Authors:** Eduardo Sampaio, Victor Rault

**Affiliations:** 1 Department of Collective Behaviour, Max Planck Institute of Animal Behavior, University of Konstanz, Konstanz, Germany; 2 Centre for the Advanced Study of Collective Behaviour, University of Konstanz, Konstanz, Germany; 3 Department of Biology, University of Konstanz, Konstanz, Germany; 4 Captain Darwin, Concarneau, France

## Abstract

Citizen-led expeditions have a unique role in fostering close connections between the public and the scientific community. In this Perspective, the authors advocate for greater support and encouragement for citizen-led projects that can help create multi-level synergistic networks for science.

## Teaser

Citizen-led explorative expeditions can foster closer connections between the public and the scientific community. Such expeditions have a considerable but mostly unrecognized track record of success and can help create important networks for advancing science.

Before the professionalization of scientific discovery, when institutions were created to directly support and fund groups of individuals to research specific areas (hereafter, researchers), the advancement of scientific knowledge mostly relied on self-motivated members of the public. In recent decades, citizen or community science has gained momentum [1,2], reaching and directly involving millions of volunteers in projects aimed at boosting conservation practices [3] or researching ecological or biological traits (e.g., [4]). However, when collaborating with researchers nowadays, the typical tasks allocated to citizens often fall within a relatively narrow spectrum of data collection and transmission [2]. Although these valuable citizen-science initiatives help increase public outreach [1] and narrow the gap between researchers and the public [2], citizens can have substantially more active functions when working alongside the scientific research community.

Despite being relatively unknown, recent endeavors led by non-governmental organizations or interested groups of citizens exploring the world’s ecosystems and biodiversity (i.e., citizen-led explorations) have led to natural history breakthroughs, helped to increase biological understanding at local and global levels, and facilitated the development and testing of overarching scientific hypotheses and theories. During the 20th century, Jacques Cousteau and his Society reported numerous natural history discoveries in their expeditions (e.g., in Le Monde Du Silence), while simultaneously pioneering technology such as the diving regulator, which has since become instrumental in the study of underwater biomes. Building on Cousteau’s explorer spirit, a recent citizen-led expedition (Under The Pole) to remote areas of French Polynesia used state-of-the-art diving equipment to find the world’s deepest living corals at 172 m [5] and, together with local scientists, verified that the incidence of coral bleaching as a result of climate change is comparatively reduced at those depths [6]. Another expedition (by Andrómede Oceanology) assisted local scientists in measuring the energetic landscape encountered by shark populations in a pristine marine reserve (Fakarava Atoll) [7], and detailed how preserving fish spawning locations can support extremely high numbers of predators, causing an inversion of the trophic pyramid [8].

Although many expeditions that explore the natural world take place in the marine realm (given its vastness and our consequently large deficit of information), some expeditions have explored remote and unusual environments on land. For example, in Le Radeau des Cîmes, a physical platform that was moved by a zeppelin around the tops of trees in tropical rainforests enabled scientists to better study the resident biota. Past scientific expeditions can also serve as models to exhort citizens to actively contribute to fundamental and overarching questions. Nearly 200 years ago, the second voyage of the HMS Beagle enabled Charles Darwin to study terrestrial and aquatic ecosystems around the world and had a crucial role in the development of the theory of evolution by natural selection [9]. Nowadays, the citizen-led expedition Captain Darwin [10] proposes to retrace the well-known route and invite onboard researchers who specialize in the scientific concepts and focus species examined by Darwin, thereby seeking to help advance our collective knowledge on these topics and convey how the visited ecosystems are changing.

Generally, expedition projects emerge when concerned citizens identify a set of skills and resources they possess that are not readily available within the scientific community, such as offshore sailing for extended timeframes (Tara Ocean), accessing remote locations like polar regions (Under The Pole) or the canopy of tropical forests (Le Radeau des Cîmes), and intercontinental mobility for sampling and special diving tools (Under The Pole, Andromède, Captain Darwin), including technical diving to >150 meters. During preparations, citizen-led explorations often cooperate with local researchers to adequately devise routes, plan sampling locations, and better examine findings. By reaching out to the scientific community, the expedition can better tailor and match the services toolbox needed for one—or several—scientific projects (Box 1). These joint projects thus leverage not only the knowledge of citizens, but also their ability to provide technical facilities and equipment for researchers to conduct their work.

**Box 1. Toolbox for researchers and citizens interested in joining or creating an expedition**

Citizen-led expeditions can be leading assets in fostering a closer relationship between the scientific community and the public. For a citizen, joining a project is a compelling way to actively help in producing scientific data and articles and improving legal tools to protect the environment. Moreover, creating or joining an expedition project can also be an opportunity to bring researchers a set of skills that can make a significant impact on their ability to produce data and communicate. For researchers, these expeditions can be valuable partners or main drivers for their research, providing logistical platforms and missing skillsets for innovative and/or complementary methodological approaches, as well as alternatives to the traditional funding sources available to the scientific community. To contextualize the design and funding of citizen-led expeditions and entice both citizens and researchers in becoming more involved, we list below examples and key features of expeditions that have been in progress for several years and are continuously recruiting new members, as well as private and public funders known for supporting these types of initiatives. We also provide hyperlinks where the respective scientific outputs and more information can be explored.

**Citizen-led expeditions**

- Tara Ocean (https://fondationtaraocean.org)
- Under The Pole (https://underthepole.org)
- Le Radeau des Cîmes (http://www.radeau-des-cimes.org)
- Captain Darwin (https://captaindarwin.org)
- Andrómede Oceanology (https://www.andromede-ocean.com)
- Jacques Cousteau Society (https://www.cousteau.org)
- Adaptation (https://www.adaptationexpe.com)

**Key features**

- Flexibility in funding options
- Unique toolbox of resources and services
- Well-defined organization and individual roles
- Continuous communication with the research, media, and public communities

**Public funding sources**

- National ministries (e.g., Education, Science)
- Regional organizations (e.g., Région Bretagne)
- Local authorities (e.g., Concarneau City)
- Non-profit organizations (e.g., National Geographic Society)

**Private funding sources**

- Private companies (e.g., Rolex, Blancpain, YEMA, Guy Cotten SAS, Patagonia, Petzl, Frisquet, BiC, L’Oréal, BNP Paribas, Honda, L’Occitane en Provence, Aqualung, Lestra, Nikon, Nauticam)
- Private foundations (e.g., EDF Group Foundation, SNCF Foundation, Crédit Agricole Foundation)

Expedition projects can be funded by a variety of sources, such as foundations in the field of science and education, private companies that often look for new ways to communicate to their target audience, and public authorities committed to funding projects that benefit their territory (Box 1). Importantly, the sources of these funds are often complementary to the scientific network of financial support (i.e., nearly no competition between scientific and expedition projects), and since expeditions are self-sufficient projects, they tend to freely offer their services to the scientific community. These additional support mechanisms provided by members of the general public can be important for researchers, particularly those in lower-income countries and remote areas where financing and/or research structures may be lacking. Citizen-led expeditions also offer unique opportunities for exploratory science in the form of outcomes that could not be predicted beforehand and are usually not favored by funding agencies in grant proposals, such as natural history findings [5,10]. Moreover, both the logistical and financial support provided by these expeditions can enable researchers to advance and expand ongoing research and objectives on overarching hypotheses, e.g., by facilitating the extension or replication of field seasons or enabling long-term manipulative in situ experiments [see 6–8].

Furthermore, not only can citizen-led expeditions provide the means for exploration and scientific work, but also they are usually leaders in the field of communication and education. Because part of their funding is often connected to foundations and private companies that need top-tier imagery for public relations, expedition projects often produce high-level photographs, films, and written stories. The knowledge of the media business and social network machinery that citizens on the expeditions possess tends to lead to a wider audience for associated researchers and scientific institutions. The same holds true in the field of education, as expedition projects often work in collaboration with schools, for which they produce specific educational material. All in all, the network of actors nurtured by expedition projects creates new synergies with the traditional scientific ecosystem.

Thus, in our society, citizen-led initiatives should be encouraged by governmental and non-governmental agencies, along with other stakeholders, to deepen and further strengthen the connection between the public and the scientific community. Beyond the traditional methods of citizen science centered on data collection and relay [1–4], citizen-led expeditions provide more prominent roles for members of the public and offer new prospects for researchers, creating a synergistic platform for the study of nature.
